# Supplementary figures and images for: PfMORC protein regulates chromatin accessibility and transcriptional repression in the human malaria parasite, Plasmodium falciparum
Source: eLife. 2024 Dec 5;12:RP92499. doi: 10.7554/eLife.92499 (PMC11620747; doi:10.7554/eLife.92499)

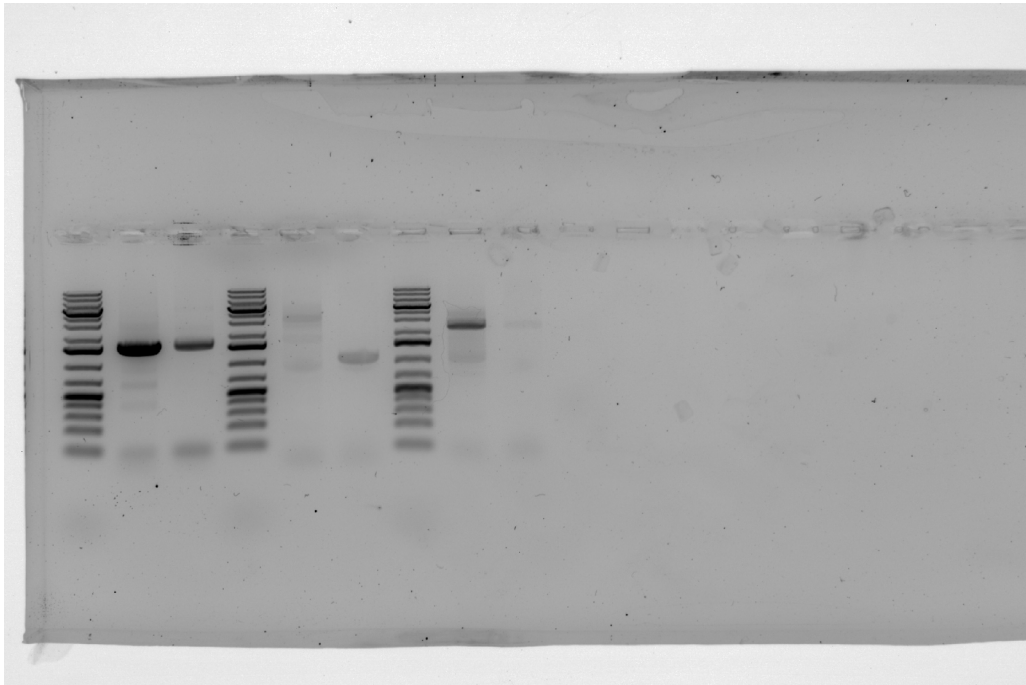

Supplement: Figure 1—source data 1. [file elife-92499-fig1-data1.zip › Figure 1-source data 1/Figure 1-source data 1.pdf]

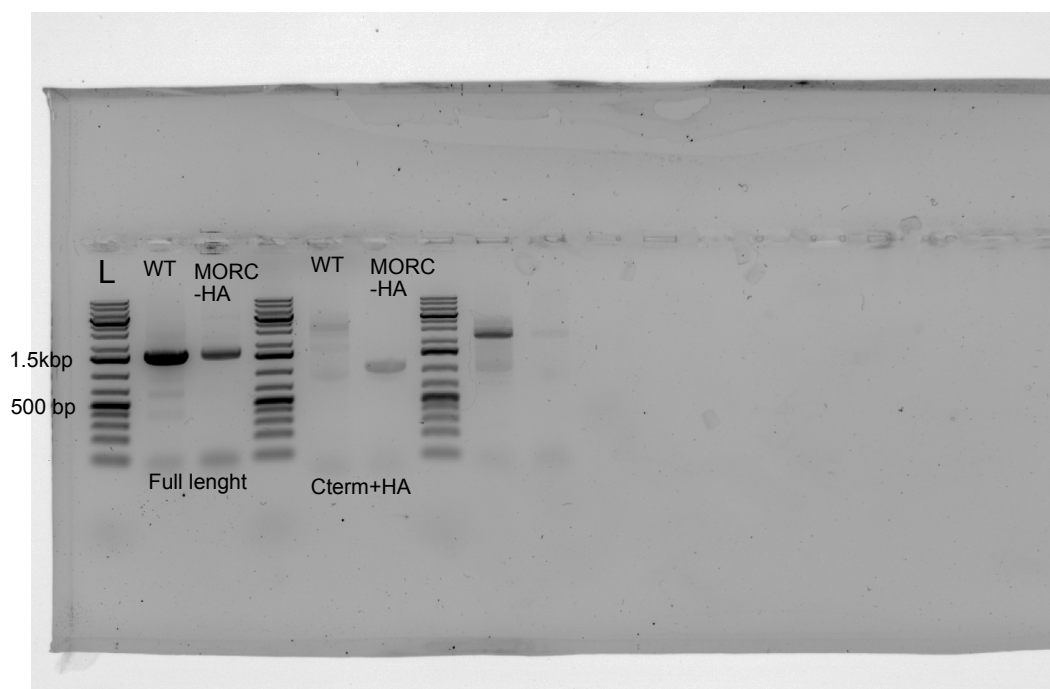

Supplement: Figure 1—source data 2. [file elife-92499-fig1-data2.zip › Figure 1-source data 2/Figure 1-source data 2.pdf]
